# Analysis of Differences in Single-Joint Movement of Dominant and Non-Dominant Hands for Human-like Robotic Control

**DOI:** 10.3390/s23239443

**Published:** 2023-11-27

**Authors:** Samyoung Kim, Kyuengbo Min, Yeongdae Kim, Shigeyuki Igarashi, Daeyoung Kim, Hyeonseok Kim, Jongho Lee

**Affiliations:** 1 Division of Advanced Science and Technology, Japan Advanced Institute of Science and Technology, Nomi 923-1292, Japan; samyoung@jaist.ac.jp; 2 Department of Brain & Neurosciences, Tokyo Metropolitan Institute of Medical Science, Tokyo 156-0057, Japan; min-kb@igakuken.or.jp; 3 Department of Computer Science and Engineering, University of Colorado Denver, Denver, CO 80204, USA; yeongdae.kim@ucdenver.edu; 4 Division of Health Sciences, Komatsu University, Komatsu 923-0961, Japan; 22212001@komatsu-u.ac.jp; 5 Department of Clinical Engineering, Kanagawa Institute of Technology, Atsugi 243-0292, Japan; kim@cet.kanagawa-it.ac.jp; 6 Swartz Center for Computational Neuroscience, Institute for Neural Computation, University of California San Diego, La Jolla, CA 92093, USA; 7 Department of Clinical Engineering, Komatsu University, Komatsu 923-0961, Japan

**Keywords:** circular tracking movement, motor control, hand dominance, prediction, visual perception

## Abstract

Although several previous studies on laterality of upper limb motor control have reported functional differences, this conclusion has not been agreed upon. It may be conjectured that the inconsistent results were caused because upper limb motor control was observed in multi-joint tasks that could generate different inter-joint motor coordination for each arm. Resolving this, we employed a single wrist joint tracking task to reduce the effect of multi-joint dynamics and examined the differences between the dominant and non-dominant hands in terms of motor control. Specifically, we defined two sections to induce feedback (FB) and feedforward (FF) controls: the first section involved a visible target for FB control, and the other section involved an invisible target for FF control. We examined the differences in the position errors of the tracer and the target. Fourteen healthy participants performed the task. As a result, we found that during FB control, the dominant hand performed better than the non-dominant hand, while we did not observe significant differences in FF control. In other words, in a single-joint movement that is not under the influence of the multi-joint coordination, only FB control showed laterality and not FF control. Furthermore, we confirmed that the dominant hand outperformed the non-dominant hand in terms of responding to situations that required a change in control strategy.

## 1. Introduction

Attempts have been made to develop robots that can imitate human movement, which involves difficult implementations. One approach focuses on unravelling the mechanism of human motor control to achieve this. While some robotic applications are designed to outperform humans, the prosthesis field, where robotic components replace human limbs, requires seamless integration with complex human motor control. In particular, both hands contribute to performing tasks effectively by a wide margin, naturally having a significant effect on robotic applications. However, to apply the mechanism of human arm control to robots, it is necessary to understand the similarities and differences between the dominant and non-dominant hands. Tasks performed with control from both arms are known to involve laterality [1,2,3,4,5,6,7,8,9]. It has been widely postulated that one cause of this phenomenon is the living environment and functional difference in the development of both arms [10,11,12,13,14].

Several previous studies on the analysis of the difference in motor control of both arms focused on multi-joint reaching tasks [15], tapping [14], and tracking tasks [16,17], and they noted that the dominant hand outperformed the non-dominant one in motor control. According to Sainburg and Kalakanis, in the goal-directed reaching movements repetitively performed by both arms, the dominant arm constructed its internal model better than the non-dominant arm [11]. In addition, it has been reported that in both two-dimensional [18] and three-dimensional tracking tasks [17], the motor control system of the dominant hand utilizes visual information more efficiently during movement, enabling precise control even at high target speeds.

In contrast, Bagesteiro and Sainburg reported that in a repetitive reaching task, the non-dominant hand exhibited better reaching performance of motor control in terms of the final position accuracy when the weight attached to the hand was heavier [12,19]. Lenhard and Hoffmann reported that in an aiming task, the non-dominant hand exhibited less constant aiming error than the dominant hand when visual information was blocked [20]. Hu et al. did not find a significant difference between both hands in terms of the time taken to move objects with various unknown weights [21].

Previous studies clearly demonstrated inconsistent differences in the motor control of both arms, as also highlighted in the results by Sainburg and Kalakanis, with a significant effect of intersegmental dynamics relevant to muscle torque control for multi-joint movement [11]. Moreover, Basgesteiro and Sainburg demonstrated the different aspects of overcompensation induced by the muscle torque between both hands using electromyographic data; the dominant hand was more likely to cause overcompensation with an increased weight, whereas overcompensation in the non-dominant hand was difficult because it had a tendency to control fewer muscles to produce power than the dominant hand [19]. These studies suggest that overcompensation may be induced by substantial agonist muscle activation during multi-joint movements [19,22,23]. The inconsistent results in motor control may be caused by the vulnerability of multi-joint tasks that are highly task-dependent, leading to different inter-joint motor coordination for each arm. Therefore, a single joint, such as the elbow or wrist, is crucial for reducing the effect of multi-joint dynamics as much as possible and accurately analyzing the difference in the motor control of both arms. Motor control was investigated in terms of feedback (FB) and feedforward (FF) controls. We adopted a manual tracking task with a sensorimotor integration system that was used in previous studies [24,25,26], which enabled us to simultaneously analyze FB and FF controls.

In this study, we examined the functional differences in cerebellar motor control between the dominant hand (DH) and non-dominant hand (NDH) in a single-joint manual tracking task. We manipulated the participants’ FF and FB controls with the visibility of the target to examine the differences in each control.

## 2. Methods

### 2.1. Participants

Fourteen participants (8 males and 6 females) with a mean age of 21.43 ± 1.1 were recruited for the experiment in this study (Table 1). All of the participants had normal or corrected-to-normal vision and were right-handed. Handedness was assessed using the Edinburgh Handedness Test. None of the participants had participated in a similar study. All of the participants provided written informed consent prior to participation. The study protocol was approved by the Ethics Committee of Komatsu University.

### 2.2. Experimental Procedure and Movement Task

**Figure 1:**
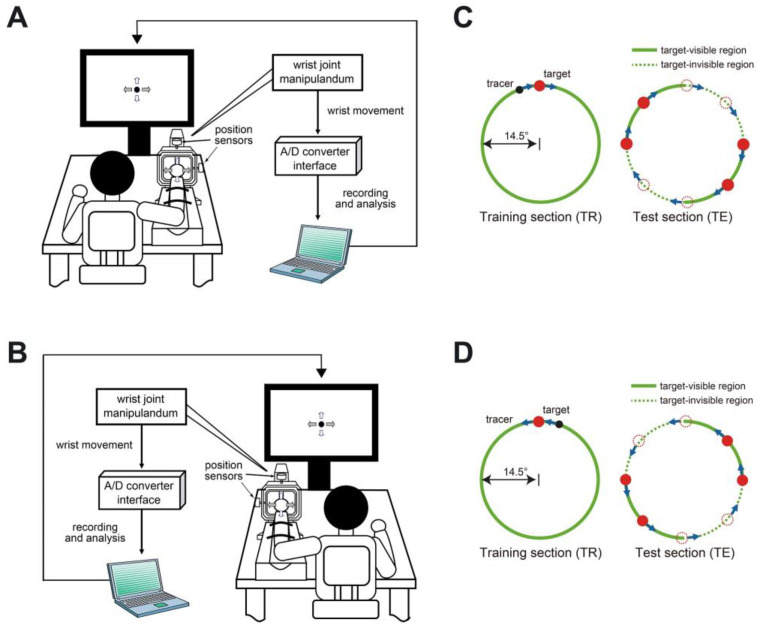
Experimental environment. (**A**,**B**) Environment apparatus. (**C**) Right-hand movement. The target moved in the clockwise direction. (**D**) Left-hand movement. The target moved in the counterclockwise direction.

We adopted a two-degree-of-freedom single-joint manual tracking task to evaluate the differences in motor control in the single-joint movement of both hands, which involved the single-joint wrist manipulation system with a laptop and an A/D converter (Figure 1A,B) used in previous studies [24,25,26]. The wrist joint manipulandum had two position sensors sampled at 1 kHz and corresponds to the tracer on the screen. The center of the manipulandum corresponds to the center of the screen. A 1° movement of the manipulandum moved the tracer by 0.66 cm. The target was represented by a red circle with a diameter of 1.5 cm. The participants were instructed to control the manipulandum and move the tracer on the screen.

Before the experiment, the participants sat on a chair 90 cm away from the monitor. The forearm was placed on a supporter such that the participants could easily hold the handle of the manipulandum. To start the task, the participants moved the tracer to a still target. Subsequently, a beep sounded three times at an interval of 1 s, and the target began to move at a constant pace, drawing a circle with a radius of 14.5 cm. The participants were asked to track the target by controlling the manipulandum.

The task consisted of two sections: training (TR) and test (TE) sections, as shown in Figure 1C,D. In the TR section, both the target and tracer were visible. They were used in order to force the participants to utilize feedback control to reduce the deviation between the target and tracer, taking advantage of the fact that a visible target can be processed with visual information and proprioception. Additionally, the TR section is where an internal model of the target’s movement is acquired through feedback error learning [27], and the internal model is stored in the cerebellum [28]. In the TE section, the target was invisible; therefore, its prediction is based on the internal model acquired in the TR section. In other words, the TE section causes FF control to compensate for the invisible target and track it properly [29,30,31,32,33,34].

The participants performed the task separately with both hands. To force the participants to use the same muscles on each side, the target moved in a clockwise direction for the right-hand task, whereas it moved in a counterclockwise direction for the left-hand task (Figure 1C,D). Therefore, the target was invisible in the first and third quadrants for the right-hand task and in the second and fourth quadrants for the left-hand task. Regarding the hand used/required to begin the task, 50% of participants commenced with their dominant hand, whereas the remaining 50% commenced with their non-dominant hand. Additionally, before the experiment began, participants were informed of which hand to perform the task with, which was determined randomly. The target moved at three different speeds with rotation periods of 12 s (tangential speed: 0.0497 m/s, referred to as speed 1), 8 s (tangential speed: 0.0746 m/s, referred to as speed 2), and 4 s (tangential speed: 0.149 m/s, referred to as speed 3). At each speed, the participants performed the task five times. The first trial was excluded from the analysis because it was used as practice.

### 2.3. Data Analysis

The angular position of the wrist joint was recorded using two potentiometers attached to the manipulandum, denoted as $tr\left(\theta_{x}\right)$ and $tr(\theta_{y})$. Accordingly, the target’s angular position which was converted from the translational position originally recorded is denoted as $TG(\theta_{x})$ and $TG(\theta_{y})$. Using these parameters, we calculated the two-dimensional position error as follows:

(1) $$position\;error=\sqrt{{\left(TG(\theta_{x})-tr(\theta_{x})\right)}^{2}+{\left(TG(\theta_{y})-tr(\theta_{y})\right)}^{2}}$$

For the statistical tests, we compared the position errors between both hands separately for FF and FB controls at three different target speeds using the two-way repeated-measure analysis of variance (ANOVA) via SPSS Statistics V.26 (IBM, Chicago, IL, USA). The following two factors were included to determine the main and interaction effects: handedness (DH and NDH) and speed (V1, V2, and V3). For ANOVA, we tested the spherical assumption; however, it was not assumed (*p* < 0.05; Mauchly’s sphericity test). Hence, the values were corrected using the Greenhouse–Geisser method. Post hoc analysis was performed using Bonferroni pairwise comparison to examine the statistical significance of the conditions for each factor. Additionally, the difference in the errors between FB and FF controls was evaluated using the paired-sample *t*-test (*t*-test function in the statistics toolbox of MATLAB Ver. 7.14.0.739(R2012a) (MathWorks, Natick, MA, USA).

## 3. Results

In this study, we examined the differences in the characteristics of the motor control of DH and NDH based on the position errors in a single-joint tracking task involving the TR section with visual information and the TE section without visual information. Fourteen participants performed the task at three different speeds with DH and NDH. The TR section was used to induce FB control, whereas the TE section was used for FF control. We denoted the target’s three speeds as V1, V2, and V3.

**Figure 2:**
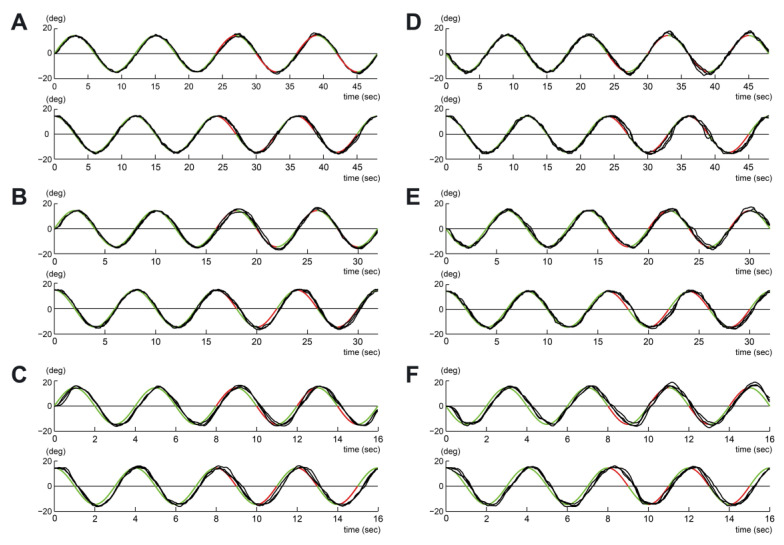
Typical example of wrist movements by the manipulandum. (**A**–**C**) correspond to the dominant hand. (**D**–**F**) correspond to the non-dominant hand. (**A**,**D**) were obtained at a tangential speed of 0.0497 m/s. (**B**,**E**) were obtained at a tangential speed of 0.0746 m/s. (**C**,**F**) were obtained at a tangential speed of 0.149 m/s.

Figure 2 shows examples of the trajectories at speed 2 (right: DH, left: NDH). The black line represents hand movement. The trajectory of the target is represented in green for the TR section and in red for the TE section for the invisible target. As shown in Figure 2, a more varying trajectory was observed in the TE section than in the TR section. Furthermore, NDH (Figure 2B) showed a more varying trajectory in both sections than DH.

**Figure 3:**
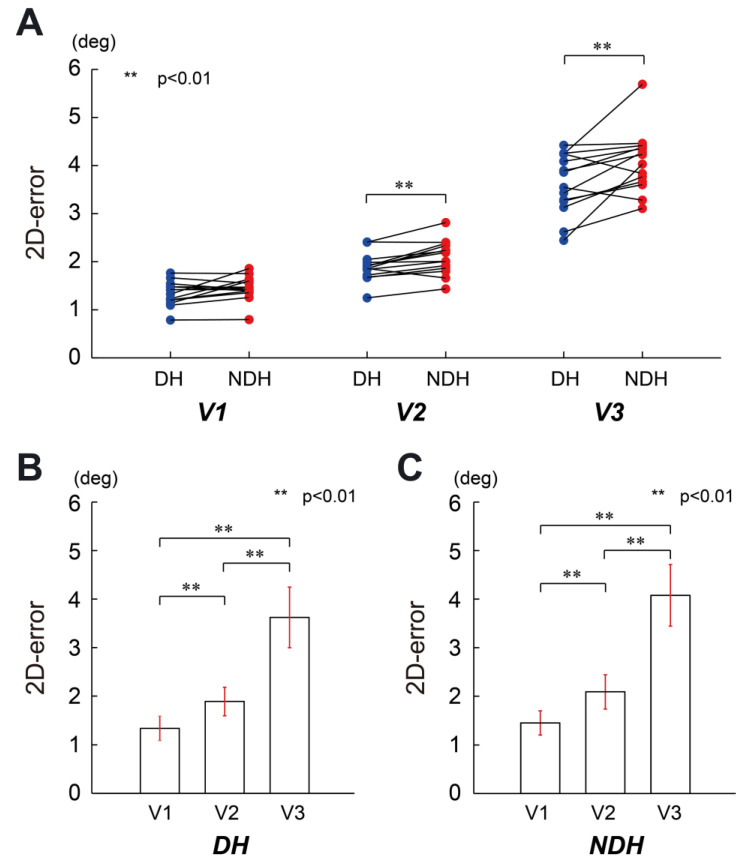
Pairwise comparison of the circular tracking position error between DH and NDH at a single joint in the FB control condition. (**A**) Pairwise comparison of the tracking accuracy errors between DH and NDH. The speeds of the target are denoted as V1, V2, and V3. (**B**) Pairwise comparison of the tracking position error for DH at different speeds. The tracking position errors are 1.34 ± 0.25 mm, 1.89 ± 0.29 mm, and 3.62 ± 0.63 mm at V1, V2, and V3, respectively. (**C**) Pairwise comparison of the tracking position error for NDH at different speeds. The tracking position errors are 1.45 ± 0.25 mm, 2.09 ± 0.35 mm, and 4.08 ± 0.64 mm at V1, V2, and V3, respectively.

First, we evaluated the position errors in FB control. Figure 3 shows a summary of the comparisons. The result of ANOVA did not indicate any significant interaction effect (F(1.231, 16.009) = 2.953, *p* = 0.10, partial $\eta^{2}$ = 0.19). On the other hand, the main effects were statistically significant for both the hand factor (F(1, 13) = 28.876, *p* < 0.01, partial $\eta^{2}$ = 0.69) and the speed factor (F(1.073, 13.947) = 489.701, *p* < 0.01, partial $\eta^{2}$ = 0.97). The post hoc analysis for the hand factor using Bonferroni correction showed significant differences in the position errors for handedness at speed 2 and speed 3 (*p* < 0.01 for both) but not at speed 1 (*p* = 0.07). The speed factor also showed significant differences between speed 1 and speed 2 (*p* < 0.01), speed 1 and speed 3 (*p* < 0.01), and speed 2 and speed 3 (*p* < 0.01) in both DH and NDH after multiple comparison corrections.

**Figure 4:**
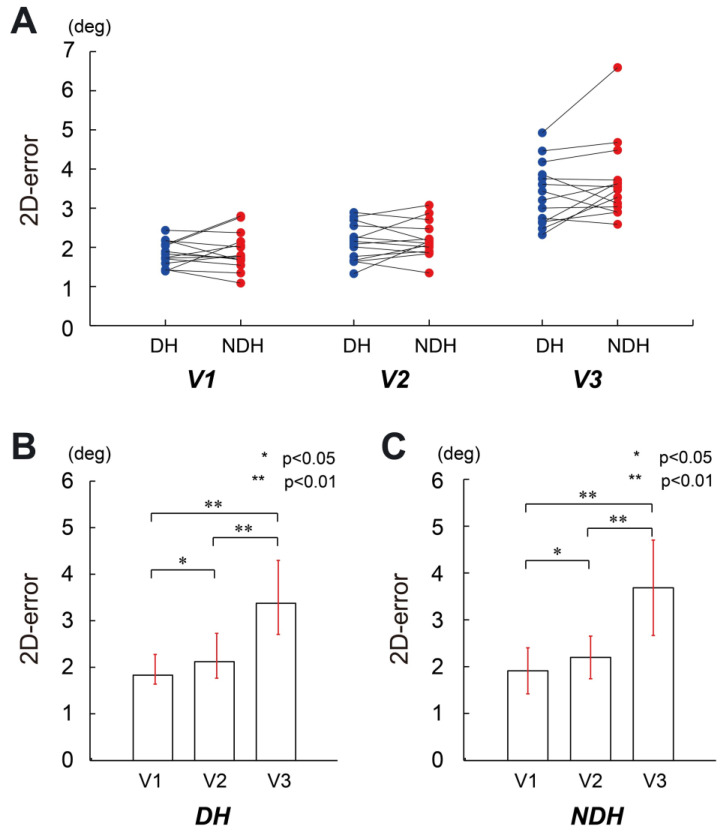
Pairwise comparison of the circular tracking position error between DH and NDH at a single joint in the FF control condition. (**A**) Pairwise comparison of the tracking accuracy errors between DH and NDH. The speeds of the target are denoted as V1, V2, and V3. (**B**) Pairwise comparison of the tracking position error for DH at different speeds. The tracking position errors are 1.83 ± 0.32 mm, 2.12 ± 0.48 mm, and 3.38 ± 0.80 mm at V1, V2, and V3, respectively. (**C**) Pairwise comparison of the tracking position error for NDH at different speeds. The tracking position errors are 1.91 ± 0.49 mm, 2.2 ± 0.46 mm, and 3.68 ± 1.02 mm at V1, V2, and V3, respectively.

Next, we examined the position errors in FF control. Figure 4 shows a summary of the comparisons. The result of ANOVA did not indicate any significant interaction effect (F(1.34, 17.47) = 1.515, *p* = 0.243, partial $\eta^{2}$ = 0.104) nor main effect of the hand factor (F(1, 13) = 2.417, *p* = 0.144, partial $\eta^{2}$ = 0.157). In contrast, we found a significant main effect of the speed factor (F(1.17, 15.23) = 57.492, *p* < 0.01, partial $\eta^{2}$ = 0.816). The post hoc analysis for the speed factor using Bonferroni correction showed significant differences between speed 1 and speed 2 (*p* < 0.05), speed 1 and speed 3 (*p* < 0.01), and speed 2 and speed 3 (*p* < 0.01) for both DH and NDH.

**Figure 5:**
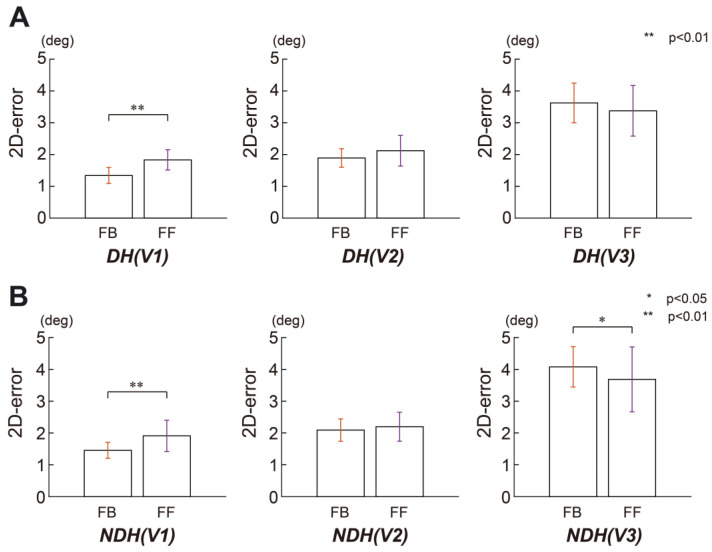
Pairwise comparison of the circular tracking position error between FB and FF at a single joint. (**A**) Pairwise comparison of the tracking accuracy errors between FB and FF for the DH condition. The speeds of the target are denoted as V1, V2, and V3. (**B**) Pairwise comparison of the tracking accuracy errors between FB and FF for the NDH condition. The speeds of the target are denoted as V1, V2, and V3.

We also examined the differences between FF and FB controls at three different speeds. Figure 5 shows the pairwise comparisons for each condition. Figure 5A shows that for DH, the difference between the FF and FB errors was significant at only speed 1 (*p* < 0.05 for speed 1, *p* = 0.12 for speed 2, and *p* = 0.18 for speed 3). In contrast, for the NDH error, the difference between FF and FB controls was significant at speed 1 (*p* < 0.05) and speed 3 (*p* < 0.05) but not at speed 2 (*p* = 0.5), as shown in Figure 5B.

## 4. Discussion

This study aimed to investigate the laterality of motor control in both upper limbs, an area still lacking a consensus. We chose wrist movements with a single-joint task to eliminate the influence of highly task-dependent multi-joint activities. We analyzed position errors in FB (TR section) and FF controls (TE section) for each hand by manipulating target visibility, with a primary focus on identifying differences in motor control between the dominant and non-dominant hands.

### 4.1. Motor Control in Single-Joint Movements

Manual tracking tasks require the integration of FF and FB controls by presenting visual information that can manipulate control strategies [29,30,31,35]. Feedback control is a closed-loop system that modifies ongoing movement with sensory information, whereas feedforward control is an open-loop system that plans movement by predicting the resultant movement based on prior knowledge and experience [26,30,36,37,38]. These FF and FB control systems are involved in maintaining accurate and efficient movement and explain the flow of neural commands. Naturally, human arms benefit from both control systems by reconciling them to adapt to spatiotemporal changes. Their interaction is considered to be a valuable clue for understanding laterality in human arms.

Our findings reveal that accuracy in both FF and FB controls decreased in the same fashion for both hands as the target speed increased (see Figure 3B,C and Figure 4B,C), indicating that the mechanisms of motor control in the task were similar. In addition, an analysis of the position errors for each section divided by the visibility of the target revealed that the dominant hand was more accurate than the non-dominant hand (see Figure 3). Our result is consistent with that of a previous study with the same parameter (angular position), where it was reported that the non-dominant hand had greater errors than the dominant hand [17]. The difference between the two arms is not considered to be a result of the excellent intersegmental dynamics of the dominant hand [11,12,39,40,41]; rather, it may be caused by different information processing capabilities for proprioception [10,17,18,42]. However, the present study, which involved single-joint movement, demonstrates that the excellent performance of the dominant hand occurred at speeds greater than 0.0746 m/s, whereas in ref. [17], the multi-joint movement showed a better performance at speeds greater than 0.149 m/s. This difference may be due to the differences in the motor torque control of single-joint and multi-joint movements, as reported by Walker [43].

For FF control, we did not observe a remarkable performance for the dominant hand (see Figure 4), suggesting that both hands have a similar capability to acquire internal models. This result is consistent with that of a previous study [44], with a tracer’s initial peak speed and the corresponding initial peak time showing no difference in FF control of the dominant and non-dominant hands. Therefore, a difference in the performance of both arms occurring in FF control [44,45] may not be attributable to the differences in muscle torque control for multi-joint movements.

### 4.2. Difference in the Transference of Motor Control for Both Arms

We compared the errors during FF and FB controls for each hand at each target speed to determine the difference in the transference of motor control for both arms at a single joint. Both arms showed significant differences in position errors at a low speed, whereas the position error in FB control was greater than that in FF control as the target speed increased. This phenomenon may not be solely due to the effect of FB control but also that of optimal feedback control, which includes both feedback control and predictive control based on a forward model (internal model) when correcting movement errors [26,46]. FB control requires approximately ten to hundreds of milliseconds for FB signals (sensory signals) to pass through the sensory system to the central nervous system. FF control works in the model to compensate for the delay in the FB signal generated as a result of a large amount of sensory information. The model is the reason for FB control working dominantly when the target was slow and for FF control working dominantly when the target was fast [31]. In our previous study on multi-joint manual tracking tasks, we suggested that the delay occurrence speed of the FB signal was 0.147 m/s or higher [37]; this result was also obtained in single-joint manual tracking tasks (see Figure 5). This suggests that the inconsistent results in motor control may not be attributable to the differences between the muscle torque control of the dominant and non-dominant hands during multi-joint movements. However, we observed that the level of compensation for the delay in the FB signal was greater in the dominant hand at a delay occurrence speed of 0.147 m/s, thereby confirming the difference in the motor control for both hands (see Figure 5).

## 5. Conclusions

This study focused on investigating the differences in motor control between dominant and non-dominant hands through single-joint tracking movements. It was confirmed that the dominant hand processed visual information more effectively using proprioception than the non-dominant hand; however, the acquisition of internal models remained the same. In addition, it was confirmed that both dominant and non-dominant hands exhibit a similar ability to respond to situations requiring movement control strategy switching, regardless of the joint type. Therefore, the inconsistent results reported in motor control may not be attributable to the differences in muscle torque control for multi-joint movements. However, a difference in the level of compensation for the FB signal delay was observed, with the dominant hand performing better. In conclusion, our study has identified distinctions in motor control between dominant and non-dominant hands. We also observed that these differences arise from functional differences in feedback signal processing. These results propose that the configuration of the feedback signal processing algorithm may differ when integrating dominant and non-dominant hands with robotic systems. We anticipate that these results will provide crucial insights into understanding the complex algorithms of feedback signals in the field of human–robot interaction, such as in prosthetics.

## Figures and Tables

**Table 1:** Participants’ information.

Participant	Age	Sex	Handedness
1	23	M	R
2	22	M	R
3	30	M	R
4	21	F	R
5	21	M	R
6	22	M	R
7	22	M	R
8	22	M	R
9	22	M	R
10	22	F	R
11	22	F	R
12	22	F	R
13	22	F	R
14	22	F	R

## Data Availability

The datasets generated during and/or analyzed during the current study are available from the corresponding author on reasonable request.
